# Reporting ethical approval in case reports and case series in 12 consecutive years: A systematic review

**DOI:** 10.1002/hcs2.113

**Published:** 2024-10-04

**Authors:** Linh Tran, Vuong Thanh Huan, Luu Lam Thang Tai, Adnan Safi, Moustafa ElBadry Ahmed, Mohamed Osman Algazar, Sedighe Karimzadeh, Nguyen Vinh Khang, Nguyen Hai Nam, Zaheer Ahmad Qureshi, Nguyen Lam Vuong, Le Huu Nhat Minh, Nguyen Tien Huy

**Affiliations:** ^1^ University of Health Sciences Vietnam National University Ho Chi Minh City Ho Chi Minh City Vietnam; ^2^ Department of Sport Medicine Pham Ngoc Thach University of Medicine Ho Chi Minh City Vietnam; ^3^ Department of Cardiology City Children Hospital Ho Chi Minh City Vietnam; ^4^ Department of Medicine Lahore General Hospital Lahore Pakistan; ^5^ Faculty of Medicine Sohag University Sohag Egypt; ^6^ Neurology Department Alharam Hospital Giza Egypt; ^7^ Department of Pharmaceutical Sciences, School of Pharmacy and Pharmaceutical Sciences University of California Irvine Irvine California USA; ^8^ Department of Neurology University Medical Center Ho Chi Minh City Vietnam; ^9^ Department of Liver Tumor, Cancer Center Cho Ray Hospital Ho Chi Minh City Vietnam; ^10^ Liver Transplant Unit, Cancer Center Cho Ray Hospital Ho Chi Minh City Vietnam; ^11^ The Frank H. Netter M.D School of Medicine at Quinnipiac University Bridgeport USA; ^12^ Department of Medical Statistics and Informatics, Faculty of Public Health University of Medicine and Pharmacy at Ho Chi Minh City Ho Chi Minh City Vietnam; ^13^ International Ph.D. Program in Medicine, College of Medicine Taipei Medical University Taipei Taiwan China; ^14^ Global Clinical Scholars Research Training Program Harvard Medical School Boston Massachusetts USA; ^15^ Institute of Research and Development Duy Tan University Da Nang Vietnam; ^16^ School of Medicine and Pharmacy Duy Tan University Da Nang Vietnam; ^17^ School of Tropical Medicine and Global Health (TMGH) Nagasaki University Nagasaki Japan

**Keywords:** case report, case series, ethical approval, declaration of Helsinki, institutional review board, informed consent

## Abstract

Our study describes the reported rate of the Institutional Review Board (IRB) approval, declaration of Helsinki (DoH), and informed consent in the case reports and case series and investigates factors associated with the ethical approval report. We searched PubMed for case reports and case series from 2006 to 2017. Annually, we obtained the first 20 articles of a case report cluster from 20 distinct publications. This analysis initially contained at least 2400 papers, with 100 papers each study design and year. Only 26 (5.4%) of 480 included studies reported IRB approval, DoH approval, and participant informed consent; 58 (12.1%) reported two out of three ethical statements (DoH, informed consent, IRB); and 151 (31.5%) reported only one, leading to nearly 245 studies (51.0%) did not report any ethical approval item. Both clusters mentioned the DoH the least. Only years, ages, ethical item types, and cluster types were associated with ethical reporting practices. This study found the serious under‐reporting of ethical practices in both case reports and case series.

## Graphical abstract

Ethical reporting practices in case reports and case series remained consistently suboptimal over a 12‐year period. Despite the importance of informed consent, institutional review board (IRB) approval, and adherence to the Helsinki Declaration, our analysis revealed low rates of reporting for these crucial elements (38.5%, 26.9% and 7.3% respectively). Furthermore, the disproportionate in informed consent reporting rate (52.3%) within IRB‐approved case series studies suggests potential misuse of IRB approval as a surrogate for obtaining patient consent.

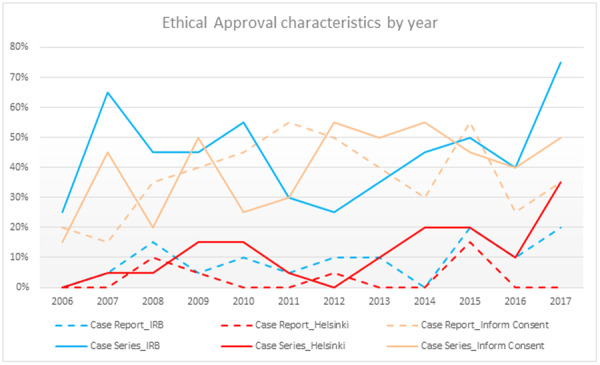

**Abbreviations**
CI: confidence interval
DoH: declaration of Helsinki
IRB: institutional review board
RCT: randomized controlled trial
REC: Research Ethics Committee

## 1 INTRODUCTION

Over the last decades, an increasing number of human research, especially in case reports and case series [1], has emphasized the important role of ethical approval [2, 3]. Ethical considerations have changed from no rule to a wide consensus with strict regulations in the clinical trials and epidemiological research for tens of years [4, 5, 6]. The concept of informed consent began with a series of judicial decisions in the early 20th century, which generated the fundamental principle of patient autonomy. However, the basis of “informed consent” remained unknown until the term was first publicly recorded in the court documents for the 1957 case [7]. An institutional review board (IRB), also known as an independent ethics committee, is an administrative unit that applies research ethics by reviewing the proposed methods to ensure that they are ethical [8]. IRB is widely required by the law or regulation in jurisdictions globally [9].

In any research that involves human participants, the authors prepared for the declaration of Helsinki (DoH) [10] to present their manuscripts to an appropriate committee called IRB [11] and to take informed consent from participants to publishers. These three items came from three different parties contributed to ethical practice and protection of the patients' rights, welfare, and health. Also, patients should anticipate the purpose, benefits, risks, and other options of the test or treatment. Meanwhile, authors not only have to gain the approval to carry out the research activities under the supervision of the affiliated institution, but also need to obtain a set of ethical standards, which was developed by the World Medical Association. Those statements of ethical principles should be entirely read and applied with consideration.

However, there exists an ever‐expanding issue, which is associated with the increasing number of publications. While most medical journals require confirmation of IRB approval and patient consent before publishing a manuscript, there is a lack of these requirements in journal publications [12, 13, 14, 15] or errors related to ethical approval [16, 17, 18]. As a result, there are raising concerns about authors' reporting integrity and patients' rights [19].

Most studies on ethical approval have been published years ago. Therefore, those publications mostly focused on randomized controlled trials (RCTs), prospective observational studies, and retrospective observational studies [20], whereas few studies carried on case reports/case series as human subjects research [21]. Acknowledging that issue, we hypothesize the high‐quality reporting of ethical components with a clear explanation in protocols and manuscripts of case reports/series, which are decent execution for journals and readers in recent years. It is noticed that no case report is published in the absence of consent [22]. Case reports/series are generally retrospective although can occasionally be prospective, which can also define their subject by exposure or outcome [23]. Case reports/series commonly contain demographic information about the patients, such as age, gender, and ethnicity. When more than three patients are included, the case series are considered as a systematic investigation to highly contribute to the research, therefore, submission is required to the IRB.

In response to these issues, we aimed to assess the frequency of reported ethical approval and informed consent. Furthermore, we also aimed to determine the correlation between characteristics and factors of study populations in reporting ethical statements throughout the last decade.

## 2 MATERIALS AND METHODS

### 2.1 Search strategy

To determine whether ethical approval has been reported in case reports and case series, we established a review process from 2006 to 2017 and initially retrieved 2400 papers (200 papers per year). To identify publications, we searched PubMed for case reports with the search terms: “Case Reports” [Publication Type] OR (“Case Reports”) and for case series with the search terms: “Case series” [Publication Type] OR (“Case series”). After rechecking inclusion and exclusion criteria and excluding duplicated papers, we randomly selected first 20 papers per study design per year from 2066 eligible papers for data extraction, resulting in 480 papers in total. In an effort to control for the journal influence on ethical approval reports, we set the additional criteria that 20 articles per year of each study design, not only were randomly selected but also must come from completely different journals to ensure our study was not focused on some journals which mainly reported case reports/case series.

### 2.2 Study selection and data extraction

Three researchers independently screened full texts of identified papers, which were fulfilled the following criteria: (1) defined as a case report or a case series while it could be a normal report, letter to editor, or clinical images reports; (2) articles with available full‐text; (3) article in English; (4) not a review, thesis, or conference; (5) human studies only; (6) no restriction was set concerning age.

The data from all included studies were extracted by three independent authors, which include the title, year of publication, ethical approval (IRB approval, DoH, informed consent), and related characteristics, and then went to consensus with the senior (Nguyen Tien Huy) if necessary. Each author must separately justify his extraction in a discussion to resolve disagreements.

### 2.3 Terms and definitions

Type of study was divided into clinical study and nonclinical study. Clinical study was defined as a study that involved with diagnosis, treatments and evaluates their effects on human health outcomes [24]. In contrast, nonclinical study related to educational cases, economical cases, techno‐engineering cases, quality and administration cases as well as ethical cases, some medical and psychological cases which did not include treatment‐related.

Type of paper described the presentation structure of the study. The common format, or the introduction, methods, results and discussion (IMRAD) structure format, was defined as a paper which was structured by four main sections: Introduction, Methods, Results, and Discussion [25]. Letter to the editor was defined as a brief paragraph to present an issue while image report was defined as obtained mainly images with few explanations.

### 2.4 Outcome measures

The primary outcome included the prevalence of reporting ethical approval (IRB approval/ethics approval/research ethics committee (REC) approval, DoH, and informed consent) in case reports/series throughout the last decades, and the comparison between case reports/series on reporting ethical approval. The secondary purpose was to determine the correlation between characteristics and factors of study populations and to report ethical approval.

### 2.5 Data analysis

SPSS statistic version 24 was used to analyze data in the study. Descriptive analysis was initially performed and expressed in terms of percentage and frequencies to describe study characteristics, along with an ethical statement reporting rate in practice. For inferential statistics, the level of significance was held at 95% confidence interval (CI) and the level of precision was chosen at 0.05. The comparison of applied proportions in practice among three ethical statement types was done by using Cochran's *Q* test and McNemar's tests. We also used Fisher's exact test to determine statistical associations between ethical statement reporting and potential factors and Mann‐Kendall trend test to evaluate the trend of reporting rates. The relationships were adjusted again using multivariable logistic regression and ordinal regression.

## 3 RESULTS

### 3.1 Search results

**Figure 1:**
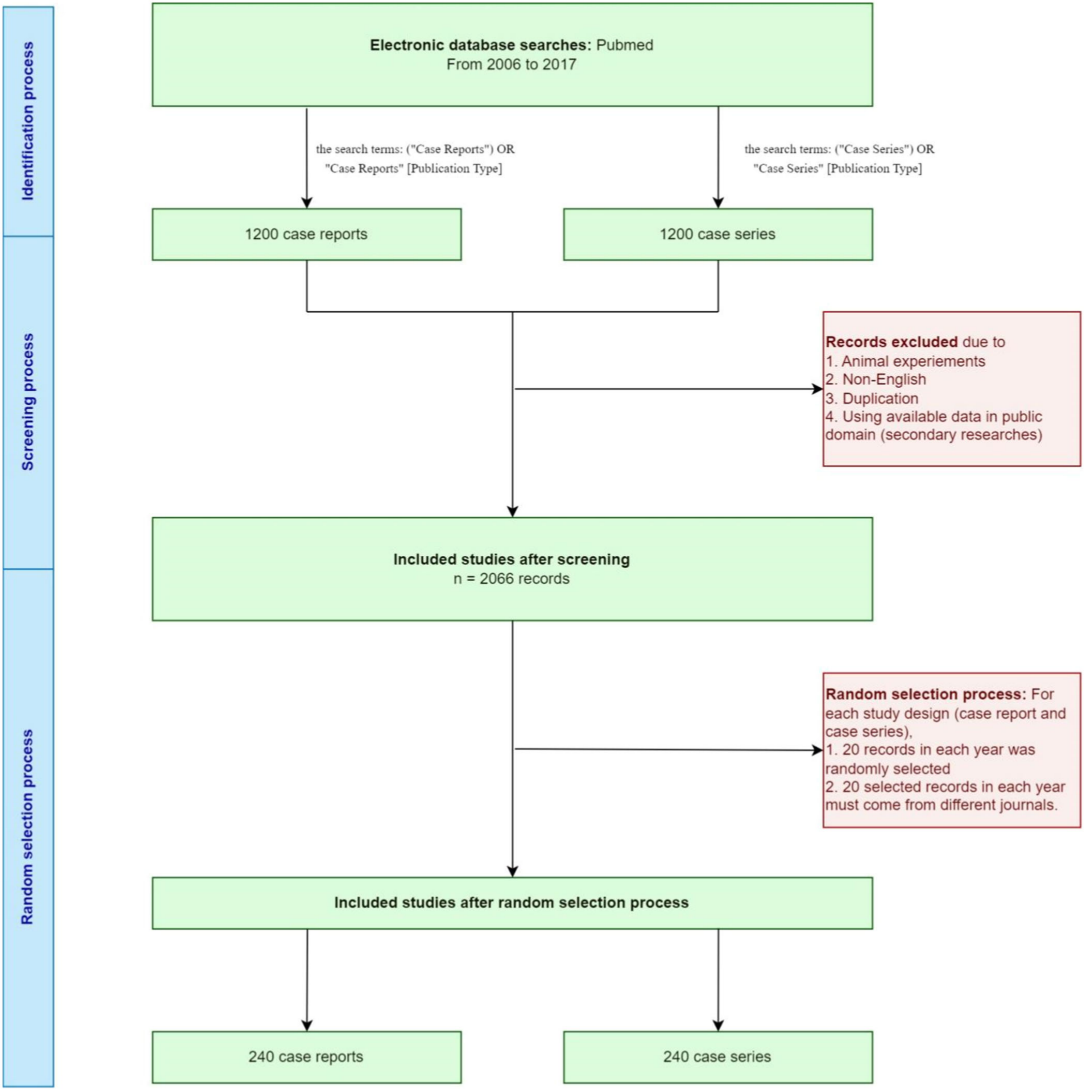
The workflow of the study.

We initially retrieved 2400 papers from the PubMed database including 1200 case reports and 1200 case series. In each cluster of case reports/series, articles were retrieved from 12 consecutive years, including 20 papers from 20 different journals each year. Following title and abstract screening, 2066 articles were eligible for full‐text screening. After checking the inclusion and exclusion criteria, 480 included studies were finally selected for data extraction. Reasons for excluding the remained studies were provided in the diagram (Figure 1).

### 3.2 Study characteristics

Characteristic description of included studies was summarized in Table 1, which included sample population, multinational research, country/continent of first authors, type of paper, and type of study. Among 480 included studies, there were 442 clinical studies (92.1%) and 38 nonclinical studies (7.9%). Regarding the target population, adults were involved in 359 studies (74.8%), while children and infants accounted for 67 studies (14.0%). In addition, 52 studies (10.8%) included both adults and children/infants and two studies did not determine the population. Regarding the type of research, 13 studies (2.7%) were clinical image reports, 17 studies (3.5%) were letters to editors and 450 articles (93.8%) were reported in a common format. Also, multinational reports were revealed in 64 studies (13.3%), whereas the remaining articles (86.7%) only investigated one country. For the distribution of the first author's continent, Europe was predominant, with 35.2% of the total included papers followed by North American (31.9%), Asian (24.2%), South American (3.1%), Australian (3.1%), and African (1.7%). When comparing between two types of study designs, case series included more clinical studies and were written in a common format more than case reports. Both types of design investigated adults as the most common population. Approaching the continent of first authors, Europe reached the highest case report (40.4%) meanwhile case series were published mostly by North American authors (38.3%). However, the top three reported continents have remained the same for both case report and case series.

**Table 1:** Description about study characteristics.

Study characteristics	Total *n* = 480 (%)	Case report *n* = 240 (%)	Case series *n* = 240 (%)	*p* value
Sample population^a^				<0.001
Adults	359 (74.8)	197 (82.1)	162 (67.5)	
Children/infants	67 (14.0)	38 (15.8)	29 (12.1)	
Both (adults + children/infants)	52 (10.8)	4 (1.7)	48 (20.0)	
Not determine	2 (0.4)	1 (0.4)	1 (0.4)	
Multinational research				0.347
Yes	64 (13.3)	28 (11.7)	36 (15.0)	
No	416 (86.7)	212 (88.3)	204 (85.0)	
Country continent of first authors^b^				0.011
Asia	116 (24.2)	63 (26.3)	53 (22.1)	
Europe	169 (35.2)	97 (40.4)	72 (30.0)	
North America	153 (31.9)	61 (25.4)	92 (38.3)	
South America	15 (3.1)	8 (3.3)	7 (2.9)	
Australia	15 (3.1)	4 (1.7)	11 (4.6)	
Africa	8 (1.7)	5 (2.1)	3 (1.3)	
Africa + Europe	1 (0.2)	1 (0.4)	−	
Europe + North America	1 (0.2)	−	1 (0.4)	
North America + South America	1 (0.2)	−	1 (0.4)	
Not determine	1 (0.2)	1 (0.4)	−	
Type of paper				<0.001
Original	450 (93.8)	216 (90.0)	234 (97.5)	
Image	13 (2.7)	13 (5.4)	0 (0.0)	
Letter	17 (3.5)	11 (4.6)	6 (2.5)	
Type of study				<0.001
Clinical	442 (92.1)	208 (86.7)	234 (97.5)	
Nonclinical	38 (7.9)	32 (13.3)	6 (2.5)	

^a^ Fisher's Exact test was performed after removing two not determine population studies.

^b^ Fisher's Exact test was performed after removing four not determine and mix continents studies.

### 3.3 Ethical statements characteristics

The ethical statement characteristics of the included studies have been described in Table 2. In overall, even being the most common ethical statement, only 185 studies (38.5%) had simultaneously reported informed consent from patient/patient's relative. The IRB approval and DoH were even reported with lower rates, respectively, 26.9% and 7.3%. To this surprising result, lack of ethical statements was dominant among 480 included articles, accounting for 51%, in general, with 62.5% and 39.6% for case reports/series, respectively. Cochran's *Q* test showed statistically a significant difference in the frequency of each ethical statement in practice, in both case reports/series (*p* < 0.001). Namely, regarding case reports, the number of articles that had informed consent (37.1%) or IRB approval (9.2%) was significantly higher than those that have DoH (2.9%). Case series' results were not much different while IRB approval (44.6%) and informed consent (40%) were predominantly and significantly available, compared with the modest numbers of reported DoH (11.7%). Nonetheless, the proportions of IRB approval in practice were slightly higher than informed consent, though there was no statistical difference found. Notably, in IRB approval studies, while almost case report studies obtained patient consent with the rate of 90.9%, only 52.3% case series did so (Supporting Information S1: Table 1).

**Table 2:** Description about ethical item statement characteristics.

Characteristics	Total *n* = 480 (%)	Case report *n* = 480(%)	Case series *n* = 480 (%)
Type of ethical statements^a^			
Having IRB approval			
Yes	129 (26.9)	22 (9.2)	107 (44.6)
No	351 (73.1)	218 (90.8)	133 (55.4)
Helsinki declaration			
Yes	35 (7.3)	7 (2.9)	28 (11.7)
No	445 (92.7)	233 (97.1)	212 (88.3)
Having informed consent			
Yes	185 (38.5)	89 (37.1)	96 (40.0)
No	295 (61.5)	151 (62.9)	144 (60.0)
Number of ethical statements for each study			
Nonethical statements	245 (51.0)	150 (62.5)	95 (39.6)
One ethical statement	151 (31.5)	70 (29.2)	81 (33.8)
Two ethical statements	58 (12.1)	14 (5.8)	44 (18.3)
Three ethical statements	26 (5.4)	6 (2.5)	20 (8.3)

Abbreviation: IRB, institutional review board.

^a^ Cochrane's *Q* test (*p* < 0.001) shown that there is statistical significant difference between ethical statements proportions in practice in each studies. In detail follow the Mcnemar test results: For case report, informed consent is statistical higher than IRB approval, and IRB approval is statistical higher than Helsinki declaration. For case series, informed consent and IRB approval are statistical higher than Helsinki declaration. The proportions of IRB approval in practice is sightly higher than informed consent, but there is no statistical significant difference.

### 3.4 Ethical approval characteristics by year

**Figure 2:**
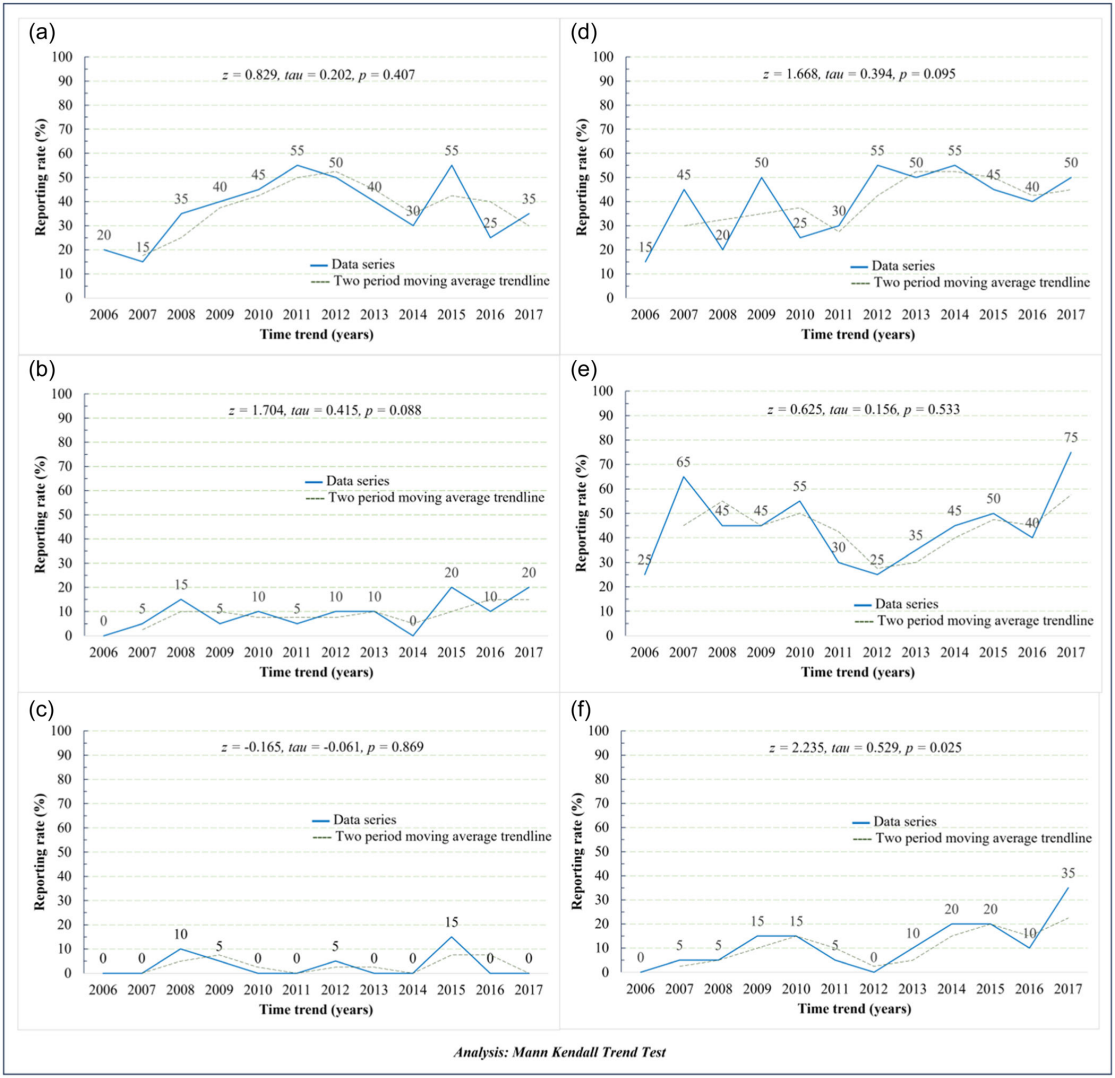
Ethical approval characteristics by year. Twelve‐year trends of ethical statements in case report and case series researches. (a) Case report informed consent obtain; (b) case report institutional review board (IRB) approval; (c) case report declaration of Helsinki (DoH); (d) case series informed consent obtain; (e) case series IRB approval; (f) case series DoH.

The change in reporting ethical statements over the years was described in Figure 2. In general, although there was variability among years, only the practice of reporting DoH in case series was expressed a significant increment by years, with Mann‐Kendall trend test *z*, *τ* being 2.235, 0.529, respectively, with *p* = 0.025.

### 3.5 Relation between ethical statements characteristics and participant population/region country of the first author

In ethical statement reporting among the type of sample population, there was a significant difference between three groups including adult, children/infant, and mixed population (included both adults and children/infant) in Table 3. When adjusting by the Bonferroni method, the articles that had a mixed population were observed with a significantly higher proportion of reporting IRB approval and DoH than those that included only an adult or children/infant. However, the proportion of informed consent reporting was approximately similar among three groups.

**Table 3:** Relationship between ethical statements characteristics and participant types (*n* = 478^a^).

Characteristics	Sample population			*p* value
	Adults only *n* = 359 (%)	Children/infants only *n* = 67 (%)	Both *n* = 52 (%)	
Type of ethical statements^b^				
IRB approval				<0.001
Yes	79 (22.0)	23 (34.3)	27 (51.9)	
No	280 (78.0)	44 (65.7)	25 (48.1)	
Helsinki				0.005
Yes	25 (7.0)	1 (1.5)	9 (17.3)	
No	334 (93)	66 (98.5)	43 (82.7)	
Informed consent				0.791
Yes	136 (37.9)	28 (41.8)	21 (40.4)	
No	223 (62.1)	39 (58.2)	31 (59.6)	
Number of ethical statements for each study^c^				0.011
Nonethical statements	191 (53.2)	33 (49.3)	19 (36.5)	
One ethical statement	117 (32.6)	17 (25.4)	17 (32.7)	
Two ethical statements	34 (9.5)	16 (23.9)	8 (15.4)	
Three ethical statements	17 (4.7)	1 (1.5)	8 (15.4)	

Abbreviation: IRB, institutional review board.

^a^ Two not determined population has been removed before performing analysis.

^b^ Fisher's Exact test.

^c^ Kruskal‐Wallis test.

For the first author's continent analysis, no statistical difference of DoH, informed consent report, and total ethical statement's number was found between separate continents. The distribution of IRB approval reports, however, was affected by continents (*p* = 0.003). Of which, the IRB report in practice was highest in North America (38.6%) and lowest in Africa (0%), although no statistical difference was found when adjusting the pairwise comparison with the Bonferroni method (Supporting Information S1: Table 2).

### 3.6 Relation between having at least one ethical statement and study characteristics

The association between study characteristics and reporting ethical statements was assessed by using binary and multivariable logistic regression analysis (Table 4). Compared with case reports/series reported at least one ethical statement more common (odds ratio [OR] = 2.3, 95% CI: 1.5–3.5, *p* < 0.001). Additionally, reporting at least one ethical statement was significantly slowly increased over the years (OR = 1.1, 95% CI: 1.0–1.1, *p* = 0.005). Furthermore, years and study design were also associated with each separate ethical statement and the total number of ethical statement reports. These results were presented in Supporting Information S1: Tables 3–6.

**Table 4:** Relation of having at least one ethical statements with study characteristics (*n* = 474^a^).

Characteristics	Univariate analysis			Multivariate analysis		
	OR	95% CI	*p* value	OR	95% CI	*p* value
Study design						
Case report	Reference	−	−	Reference	−	−
Case series	2.575	1.778–3.729	<0.001	2.274	1.500–3.448	<0.001
Year	1.083	1.027–1.141	0.003	1.083	1.024–1.145	0.005
Sample population						
Adults	Reference	−	−	Reference	−	−
Children/infants	1.173	0.696–1.978	0.549	1.260	0.708–2.240	0.432
Both	1.977	1.083–3.609	0.026	1.379	0.709–2.682	0.344
Multinational research						
Yes	1.579	0.915–2.725	0.101	1.626	0.905–2.922	0.104
No	Reference	−	−	Reference	−	−
Country continent of first authors						
Europe	Reference	−	−	Reference	−	−
Asia	1.293	0.805–2.078	0.288	1.115	0.673–1.846	0.672
North America	1.219	0.786–1.892	0.377	1.032	0.648–1.644	0.896
South America	0.563	0.185–1.719	0.313	0.388	0.117–1.283	0.121
Australia	0.751	0.256–2.204	0.602	0.526	0.172–1.608	0.260
Africa	0.376	0.074–1.914	0.239	0.309	0.058–1.649	0.169
Type of paper						
Original	3.082	0.823–11.534	0.095	2.525	0.639–9.980	0.186
Letter	1.000	0.178–5.632	1.000	0.939	0.160–5.493	0.944
Image	Reference	−	−	Reference	−	−
Type of study						
Clinical	1.650	0.828–3.292	0.155	1.372	0.648–2.904	0.409
Nonclinical	Reference	−	−	Reference	−	−

Abbreviations: CI, confidence interval; OR, odds ratio.

^a^ Studies which cannot determine sample population or continent of authors, and studies which authors came from mutiple country continents were removed before performing analysis.

### 3.7 Relation of having IRB approval with study characteristics

Univariable logistic regression demonstrated that “case series” in study design, included “children/infant” or “both children/infant and adult” in the sample population, “multinational characteristic” in research, “North America” of first author's continent, “common format” in the type of paper was linked to increasing the chance of having IRB approval. However, through multivariable regression analysis, “common format” in type paper was not considered as a significantly associated factor, whereas other remaining items were the determinant of their statistical impact.

### 3.8 Relation of having DoH with study characteristics

Univariable logistic regression demonstrated that “case series” in study design, including “both children/infant and adult” in the sample population and “year” were associated with an increase in the chance of having DoH. However, after including significant factors in univariable analysis for multivariable regression analysis, we only found that “case series” and “year” were proved as significant predictors.

### 3.9 Relation of having informed consent with study characteristics

Univariable logistic regression did not prove any significant factor that contributed to the chance of having informed consent. However, in the multivariable model, North America surprisingly seems to be the least reported in informed consent.

### 3.10 Checklist practice in case reports/series

In a wider aspect, we evaluated the practice of the CARE (CAse REport) checklist [26] for the case report's cluster. This checklist gauges the report's quality by yes/no questions about the existence of important must‐have items available for a report publication, including ethical approval items. In the score scale, we calculated how many “yes” from the 30 items in the CARE checklist that the reports obtained so that we could compare the quality between reports. Finally, we calculated the average scores (number of “yes”) of 20 papers each year from 2006 to 2017 (Supporting Information S1: Figure 1). Overall, the score generally experienced a downward trend between 2006 and 2017. The score reached a peak in 2010 (22.05 points), and in 2016 (16.80 points) registered a substandard performance compared to the other years. Although the score of 22.05 was the highest average, containing nearly three‐fourth of the must‐have items, the inferior one (16.80) obtained slightly more than half of the required items.

Likewise, in the cluster of case series, we assessed the quality based on the Cancer Council Australia's checklist [27] which has only three questions divided into three levels: low, moderate, and high risk of bias. From that, we calculated the number of papers in each risk‐level group for each year. Startlingly, 4 years (2008, 2009, 2010, and 2013) collapsed with all high‐risk papers (20/20) (Supporting Information S1: Figure 2). However, the last 4 years' outcome (from 2014 to 2017) accelerated remarkably when over 50% of papers each year were moderate‐risk while few papers reached a low‐risk level. This could raise the anticipation of ethical approval practice was on the pace of improvement.

## 4 DISCUSSION

Along with the increasing numbers in the case reports/series, our study found that reporting of IRB approval, DoH, and informed consent had not reached the expected intensive level.

Among 480 included articles, more than half of included studies surprisingly lacked ethical statements, most of them did not fulfill all three components of ethical approval. Meanwhile, there was a significant difference in frequency between ethical components that persisted from case reports/series. The Cochran's *Q* test statistically presented a significant difference between the frequency of informed consent (37.1%), IRB approval (9.2%), and DoH (2.9%).

Realizing that previous reviews did not have sufficient attention on ethical approval in case reports/series, we investigated the studies working on RCTs only or a mixture of study designs. In detail, Schroter et al. [28] previously reported among five famous medical journals, namely *Annals of Internal Medicine*, *BMJ*, *JAMA*, *Lancet*, and *The New England Journal of Medicine*, stated that ethical approval and informed consent were not mentioned in 31% and 47% of 350 included articles, respectively. Meanwhile, a lack of reporting in both ethical approval and informed consent was presented in 88 (27%) manuscripts. The ethical practice outcome of Schroter's study was slightly higher than our results, which could be explained by the inclusion criteria, which were all types of study designs. Because the other study designs were frequently reported as a better ethical practice than case reports/series [29]. The study of Myles and Tan [29] investigated reporting ethical approval and informed consent of four anesthesia journals, in a wide range of study designs (1189 studies). A similar outcome to our study was presented when the authors pointed out that case reports/series were the least designed to include IRB approval and consent. IRB approval in case reports/series was obtained in only 2% meanwhile RCTs and mechanistic studies had more than 90% for each. Similar to the informed consent, case reports/series only have 3%, compared to RCTs and mechanistic studies with 97% and 83%, respectively. To support the earlier results, another study [30] working on more than 500 articles from 54 issues of the four surgery journals reported similar statistics to our study that reports (67%) did not include any statement on human subject protection. Only 74 reports (14%) mentioned both ethical approval and informed consent. Dingemann et al. [31] also reported the lowest rate, only 1.4% reporting ethical approval, and 1% informed consent was observed in 142 case reports. Moreover, corresponding to the significant difference between ethical components' recurrence in our study, two studies [32, 33] also reported that rates of IRB approval and informed consent in medical journals have improved better than DoH over the two previous decades. In a silver lining, our recent study [34] on only RCTs ethical approval showed that among 927 included trials, only 1.5% of these are lacking the ethical statements. Yet, nearly two‐third (63%) of clinical trials did not completely report the investigated components (informed consent, IRB approval, and DoH).

In short, previous studies and our study have pointed out some features in common: ethical approval practice in case reports/series is not well mentioned as RCTs and the other research types meanwhile DoH is the least component that was documented in reports. Besides, limited studies have worked on the characteristics and factors of study populations, thus we could not compare to our study. However, in our study, the characteristics and factors of study populations and reporting ethical approval also presented a minor correlation as expected.

There were plenty of reasons for not seeking ethical approval in case reports/series, some studies [15, 35] demonstrated that ignorance, perceptions of cost, perceptions of certain research being exempt, impossibility to take consent (i.e., because of incompetence or age, emergency), no apparent and intelligible universal consensus. A further important question clearly concerns why the frequency of DoH is not high, in comparison with the two other components. The answer may come from the direct protection of the human subject's role of informed consent and IRB approval [22, 29], which are considered the major components. Moreover, the DoH is a set of ethical principles that serves as a guiding framework rather than a specific, detailed criteria checklist [36]. There also remains a considerable controversy about the DoH [37], along with the complicated and demanding protocols, which makes for some inevitable missing report of this component. Nevertheless, DoH itself has widely changed to update the variety of research forms and it still has a definite value when paying awareness of the need to facilitate research and the need to protect vulnerable patients [38]. In addition, IRB approval and DoH, the articles that included both adults and children/infants were significantly higher than those that included solely adults or children/infants. We believe that this is a remarkable point for the population when researchers want to conduct reports or trials: the younger the population, the more thoughtfulness of ethical approval should be made to reach the best treatment condition for the patients and the quality of the study.

Unlike the DoH, the issue of informed consent in case reports and case series has been a concern for a long time, to balance the right of patient confidentiality and the benefits to medicine and society at large coming from sharing their stories. In 1995, an international committee of 12 biomedical journal editors finalized guidelines for publishing case reports with the principle that patient privacy should take precedence over the need for clinicians to contribute knowledge and experience [39]. Since the 1995 consensus, more detailed and specific consensus on informed consent in case reports and case series have been developed over time, namely the guidelines of the Committee on Publication Ethics in 2013 [40] and the consensus of the International Committee of Medical Journal Editors in 2019 [41] with the same principle. Even though the informed consent report has been raised by time, only 38.5% of articles reported informed consent from 2006 to 2017 shown the fact that patient consent might not be taken adequately enough. The importance of obtaining patient consent if not taken seriously enough may over time cause the patient to feel violated or exploited by the doctor [42].

In addition to obtaining the consent of each patient, the publication of a case report or a series of cases may also require the approval of an IRB. In general, there are some differences in ethical considerations between case report and case series. For the case report study, there are many reasons resulting in the perforation of informed consent in ethical consideration, presented with the higher report rate compared to IRB approval in case report in our study (37.1% and 9.2%). Foremostly, most institutions do not consider case report to constitute research, leading to IRB approval is usually not required or mandatory [43]. Another reason is that even with local IRB approval, patient consent is still essential as a review criterion in many journals [40]. In contrast, IRB approval appears to be almost a mandatory requirement at many institutions before initiating research, resulting in roughly equal rate of IRB approval and informed consent (44.8% and 40.2%). However, for whatever reason, IRB approval cannot be a substitute for patient consent. Approval from an ethics committee should not overcome a patient's refusal to provide consent and whenever possible, individual patient consent should be obtained, even for retrospective research. For that reason, using an IRB as an option to waive the requirement for patient consent is another issue that needs to be concerned in research practice. Interestingly, our results found that among IRB approval studies, informed consent was only reported in 52.3% case series, compared to 90.9% in case report. That raises the question of whether IRB is a convenient form of avoiding the need for authors to obtain patient consent.

Raising the concern of ethical issues for case reports/series in the last decade, we also investigate two specific types which are considered as case reports/series: letters to the editor [44] and image cases. In these types, patients' information is also published directly for worldwide learning and discussion, sometimes their faces, injured areas on their bodies, their identities, family genealogy, and the likes are also published without permission. The common feature of these two types is short length. Unlike other normal case reports/series, letters to the editor and case images are often presented in very few pages with some brief information about the case. Some image cases contain only images and a few explanatory sentences. Although there is no relation between these two types and ethical practice, also there is no consensus or protocol to guide how to present ethical approval in these types, we assume that those types need ethical practice and we predict that these types are in high rate of missing ethical approval.

One of the limitations is that we used PubMed as only data source. Although this is one of the biggest research databases, taking other databases would be better for the quality of the study outcome. Besides, in the study process, we detected some reports on physically or mentally incapable of giving consent to patients that the legally allowed representative session of DoH did not recount. This issue could partially help to explain why several reports neglected the DoH. Like DoH, IRB approval had several similar limitations that they did not get a final consensus. Taking the case of some countries and areas, it is not compulsory for reports with the number of objects less than [45], therefore, authors easily omit this ethical component. However, IRB approval seemed to try hard to improve its quality (invite the third party) [19] and accessibility (shorten time on IRB, explain clearly the items) [46]. Our study also found a special situation in reports of forensic science which impossible to fetch ethical approval. Otherwise, the definition of case series is needed to operate when it exists in many definitions [47, 48], which will make authors and researchers confused to defining their types of publication. Further work is needed to determine the extent to which our results reflected the ethical issues. There are also considerable differences among countries [30], especially between the developing and developed countries [15, 49, 50] notwithstanding a clear protocol or consensus in journals as well as continents.

Our study focused on case reports/series because of their enormous numbers in publications. To protect the rights and safety of patients as well as the quality of research, our aspiration is not only limited to reports but also the other research types, the more researchers and editors pay attention to ethical approvals in case reports/series, the more concerns should be paid on other clinical trial's research. Accordingly, authors must observe the ethical approach, defined by the consensus, guidelines, and journal demands. In this situation, editors should play a gatekeeper role to ensure patients' rights and benefits, sustain human research's integrity, and gain public trust [51]. With the awareness of an ethical issue of every level of the research area, researchers can be best supported to avoid making errors. There must be some recommendations to prevent unethical research from being published [12] to establish and maintain the attitude and regularity in ethical practices. Furthermore, there is a necessity for a detailed and accessible consensus in the community of researchers and journal editors, including each ethical component and all types of study, particularly in reports and trials. Another limitation of this study is paper selection due to massive publication without proper categories to focus on any specific area. Hence, in an effort to control for the journal confounder, we set the additional criteria that 20 articles per year of each study design, not only were randomly selected but also must come from completely different journals. Finally, while this study focused on the trend and progress of ethics at the time of conducting this study, the authors aimed to raise the awareness of other authors and editors on the mentioned ethical approvals when conducting and approving clinical research. This study reflected a short period of time while further studies will be needed for real‐time and up‐to‐date publications worldwide.

## 5 CONCLUSIONS

Our study found an infrequent using the ethical practices in case reports/series, which is significantly lower than other study designs. More than half of included papers in our study lack of the ethical statements (DoH, IRB, and informed consent) whereas the correlation between characteristics and factors of the study population was not noticeable. Accordingly, the awareness of ethical approval in human research should be taken into consideration. There must be an imperative need to improve the situation, which comes from participants involved in medical research.

## AUTHOR CONTRIBUTIONS

**Linh Tran**: Conceptualization; data curation; validation; visualization; writing—original draft; writing—review and editing. **Vuong Thanh Huan**: Conceptualization; data curation; validation; visualization; writing—original draft; writing—review and editing. **Luu Lam Thang Tai**: Methodology; investigation; data analysis and curation; writing—review and editing. **Adnan Safi**: Data curation; methodology; validation. **Moustafa ElBadry Ahmed**: Methodology; investigation. **Mohamed Osman Algazar**: Methodology; investigation. **Sedighe Karimzadeh**: Investigation. **Nguyen Vinh Khang**: Methodology. **Nguyen Hai Nam**: Investigation; methodology. **Zaheer Ahmad Qureshi**: Validation. **Nguyen Lam Vuong**: Data curation. **Le Huu Nhat Minh**: Methodology. **Nguyen Tien Huy**: Conceptualization; visualization; validation; writing—review and editing; supervision.

## CONFLICT OF INTEREST STATEMENT

The authors declare no conflict of interest.

## ETHICS STATEMENT

Not applicable.

## INFORMED CONSENT

Not applicable.

## Supporting information

Supporting information.

## Data Availability

Data derived from public domain resources.
