# Supplementary material for: Reporting ethical approval in case reports and case series in 12 consecutive years: A systematic review
Source: Health Care Sci. 2024 Oct 4;3(5):298–311. doi: 10.1002/hcs2.113 (PMC11520241; doi:10.1002/hcs2.113)
Supplement: Supplementary file 1 — Supporting information. [file HCS2-3-298-s001.doc]

# Supplementary files

**Supplemental figure 1. Description of checklist of included case report studies from year 2006 to 2017**


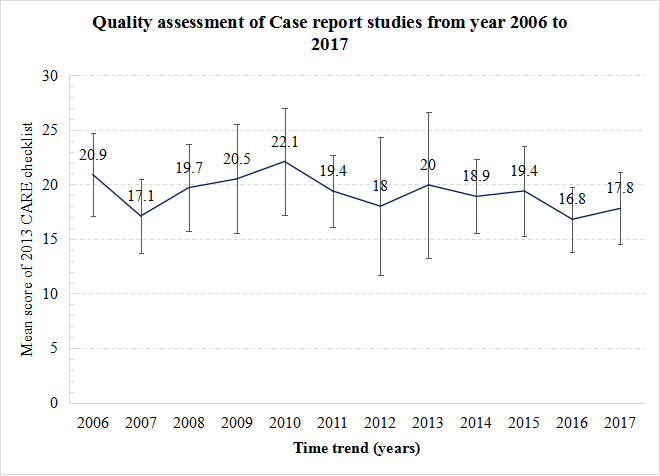


**Supplemental figure 2. Description of checklist of included case series studies from year 2006 to 2017**


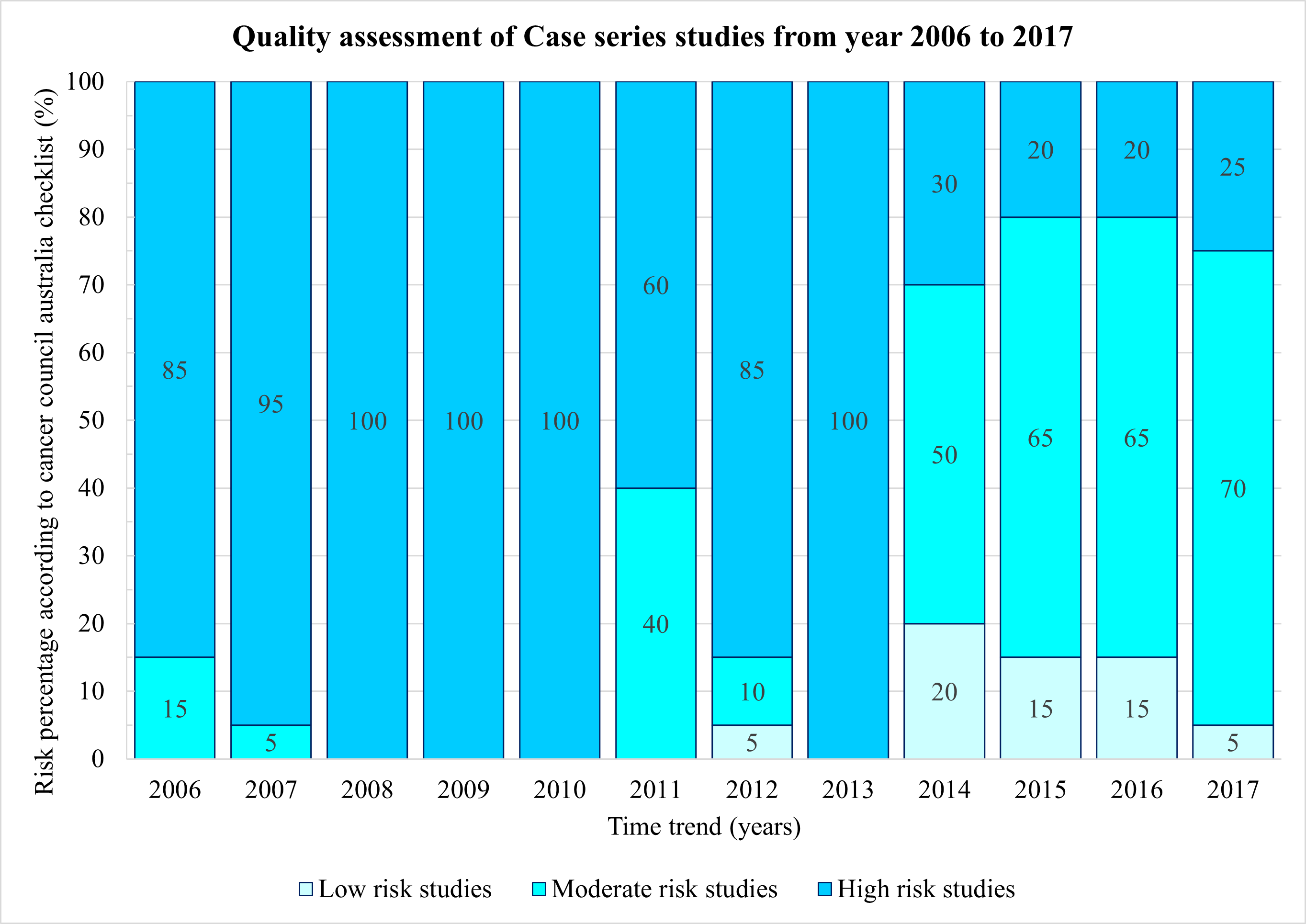


**Supplementary Table 1. Subgroup analysis - Informed consent reported in case report and case series that obtained IRB approval**

| **Characteristics** | | **IRB approval**  **(*n* = 129)** |
| --- | --- | --- |
| **Case report** | Informed consent reported | 20 (90.9) |
|  | No informed consent reported | 2 (9.1) |
| **Case series** | Informed consent reported | 56 (52.3) |
|  | No informed consent reported | 51 (47.7) |

**Supplementary Table 2.** **Relationship between ethical statements characteristics and Continent of first author (*n*=476*)**

| **Characteristics** | **Asia** | **Europe** | **North America** | **South America** | **Australia** | **Africa** | ***p* value** |
| --- | --- | --- | --- | --- | --- | --- | --- |
|  | **(*n*=116)** | **(*n*=169)** | **(*n*=153)** | **(*n*=15)** | **(*n*=15)** | **(*n*=8)** |  |
| IRB approval^a^ | 27 (23.3) | 34 (20.1) | 59 (38.6) | 4 (26.7) | 4 (26.7) | 0 (0.0) | ***0.003*** |
| Helsinki^a^ | 10 (8.6) | 15 (8.9) | 7 (4.6) | 1 (6.7) | 1 (6.7) | 0 (0.0) | 0.618 |
| Inform consent^a^ | 51 (44.0) | 72 (42.6) | 49 (32.0) | 5 (33.3) | 5 (33.3) | 2 (25.0) | 0284 |
| Number of ethical statements^b^ |  |  |  |  |  |  | 0.590 |
| Non ethical statements | 54 (46.6) | 90 (53.3) | 74 (48.4) | 10 (66.7) | 9 (60.0) | 6 (75.0) |  |
| One ethical statements | 45 (38.8) | 50 (29.6) | 49 (32.0) | 1 (6.7) | 3 (20.0) | 2 (25.0) |  |
| Two ethical statements | 10 (8.6) | 16 (9.5) | 26 (17.0) | 3 (20.0) | 2 (13.3) | 0 (0.0) |  |
| Three ethical statements | 7 (6.0) | 13 (7.7) | 4 (2.6) | 1 (6.7) | 1 (6.7) | 0 (0.0) |  |
| *(*) Studies which can not determine continent of authors, and studies which authors came from mutiple country continents were removed before performing analysis.*  *a : Fisher’s Exact test*  *b : Kruskal Wallis test* | | | | | | | |

**Supplementary Table 3. Relation of having IRB approval with study characteristics (*n*=474*)**

| **Characteristics** | **Univariate Analysis** | | | **Multivariate Analysis** | | |
| --- | --- | --- | --- | --- | --- | --- |
|  | **OR** | **95% CI** | ***p* value** | **OR** | **95% CI** | ***p* value** |
| **Study design** |  |  |  |  |  |  |
| Case report | reference | — | — | reference | — | — |
| Case series | 7.908 | 4.758–13.142 | <**0.001** | 6.847 | 3.876–12.093 | <**0.001** |
| **Year** | 1.052 | 0.992–1.116 | 0.091 | 1.071 | 1.000–1.147 | **0.050** |
| **Sample population** |  |  |  |  |  |  |
| Adults | reference | — | — | reference | — | — |
| Children/Infants | 1.856 | 1.057–3.261 | **0.031** | 2.178 | 1.102–4.303 | **0.025** |
| Both | 3.835 | 2.107–6.983 | <**0.001** | 2.286 | 1.168–4.472 | **0.016** |
| **Multinational research** |  |  |  |  |  |  |
| Yes | 2.656 | 1.530–4.611 | **0.001** | 2.732 | 1.406–5.310 | **0.003** |
| No | reference | — | — | reference | — | — |
| **Country continent of**  **first authors** |  |  |  |  |  |  |
| Europe | 0.951 | 0.400–2.262 | 0.910 | 1.532 | 0.591–3.971 | 0.380 |
| Asia | 1.138 | 0.467–2.773 | 0.777 | 1.515 | 0.567–4.050 | 0.407 |
| North America | 2.379 | 1.022–5.540 | **0.044** | 3.507 | 1.365–9.007 | **0.009** |
| Others | reference | — | — | reference | — | — |
| **Type of paper** |  |  |  |  |  |  |
| Original | 5.119 | 1.197–21.886 | 0.028 | 3.162 | 0.683–14.648 | 0.141 |
| Non-original | reference | — | — | reference | — | — |
| **Type of study** |  |  |  |  |  |  |
| Clinical | 1.641 | 0.702–3.835 | 0.253 | 0.910 | 0.326–2.540 | 0.858 |
| Non-clinical | reference | — | — | reference | — | — |
| *(*) Studies which can not determine sample population or continent of authors, and studies which authors came from mutiple country continents were removed before performing analysis.* | | | | | | |

**Supplementary Table 4. Relation of Helsinki Declaration with study characteristics (*n*=474*)**

| **Characteristics** | **Univariate Analysis** | | | **Multivariate Analysis** | | |
| --- | --- | --- | --- | --- | --- | --- |
|  | **OR** | **95% CI** | ***p* value** | **OR** | **95% CI** | ***p* value** |
| **Study design** |  |  |  |  |  |  |
| Case report | reference | — | — | reference | — | — |
| Case series | 5.158 | 2.094–12.704 | **<0.001** | 4.864 | 1.804–13.120 | **0.002** |
| **Year** | 1.153 | 1.035–1.285 | **0.010** | 1.163 | 1.035–1.306 | **0.011** |
| **Sample population** |  |  |  |  |  |  |
| Adults | reference | — | — | reference | — | — |
| Children/Infants | 0.209 | 0.028–1.572 | 0.128 | 0.177 | 0.023–1.395 | 0.100 |
| Both | 2.887 | 1.260–6.616 | **0.012** | 1.742 | 0.709–4.278 | 0.226 |
| **Multinational research** |  |  |  |  |  |  |
| Yes | 0.896 | 0.304–2.637 | 0.842 | 1.069 | 0.342–3.342 | 0.908 |
| No | reference | — | — | reference | — | — |
| **Country continent of**  **first authors** |  |  |  |  |  |  |
| Europe | 1.765 | 0.386–8.064 | 0.464 | 2.533 | 0.509–12.595 | 0.256 |
| Asia | 1.698 | 0.355–8.118 | 0.507 | 1.901 | 0.369–9.779 | 0.442 |
| North America | 0.869 | 0.173–4.362 | 0.865 | 0.996 | 0.184–5.408 | 0.996 |
| Others | reference | — | — | reference | — | — |
| **Type of paper** |  |  |  |  |  |  |
| Original | 2.157 | 0.284–16.38 | 0.457 | 0.856 | 0.102–7.158 | 0.886 |
| Non-original | reference | — | — | reference | — | — |
| **Type of study** |  |  |  |  |  |  |
| Clinical | 2.941 | 0.391–22.133 | 0.295 | 0.970 | 0.115–8.200 | 0.978 |
| Non-clinical | reference | — | — | reference | — | — |
| *(*) Studies which can not determine sample population or continent of authors, and studies which authors came from mutiple country continents were removed before performing analysis.* | | | | | | |

**Supplementary Table 5. Relation of having inform consent with study characteristics (*n*=474*)**

| **Characteristics** | **Univariate Analysis** | | | **Multivariate Analysis** | | |
| --- | --- | --- | --- | --- | --- | --- |
|  | **OR** | **95% CI** | ***p* value** | **OR** | **95% CI** | ***p* value** |
| **Study design** |  |  |  |  |  |  |
| Case report | reference | — | — | reference | — | — |
| Case series | 1.153 | 0.797–1.668 | 0.451 | 1.157 | 0.763–1.754 | 0.492 |
| **Year** | 1.053 | 0.993–1.117 | ***0.015*** | 1.069 | 1.011–1.13 | 0.019 |
| **Sample population** |  |  |  |  |  |  |
| Adults | reference | — | — | reference | — | — |
| Children/Infants | 1.170 | 0.688–1.989 | 0.562 | 1.172 | 0.669–2.052 | 0.579 |
| Both | 1.104 | 0.610–1.999 | 0.744 | 0.974 | 0.513–1.852 | 0.936 |
| **Multinational research** |  |  |  |  |  |  |
| Yes | 1.109 | 0.642–1.918 | 0.710 | 1.152 | 0.654–2.027 | 0.624 |
| No | reference | — | — | reference | — | — |
| **Country continent of**  **first authors** |  |  |  |  |  |  |
| Europe | reference | — | — | reference | — | — |
| Asia | 1.046 | 0.649–1.686 | 0.853 | 0.931 | 0.571–1.517 | 0.773 |
| North America | 0.634 | 0.402–1.002 | 0.051 | 0.598 | 0.374–0.956 | **0.032** |
| South America | 0.667 | 0.218–2.035 | 0.476 | 0.574 | 0.182–1.806 | 0.342 |
| Australia | 0.667 | 0.218–2.035 | 0.476 | 0.619 | 0.200–1.919 | 0.406 |
| Africa | 0.444 | 0.087–2.267 | 0.329 | 0.382 | 0.073–1.996 | 0.254 |
| **Type of paper** |  |  |  |  |  |  |
| Original | 1.974 | 0.527–7.392 | 0.313 | 2.119 | 0.544–8.249 | 0.279 |
| Letter | 1.000 | 0.178–5.632 | 1.000 | 1.070 | 0.186–6.155 | 0.939 |
| Image | reference | — | — | reference | — | — |
| **Type of study** |  |  |  |  |  |  |
| Clinical | 1.046 | 0.524–2.089 | 0.899 | 1.120 | 0.535–2.342 | 0.763 |
| Non-clinical | reference | — | — | reference | — | — |
| *(*) Studies which can not determine sample population or continent of authors, and studies which authors came from mutiple country continents were removed before performing analysis.* | | | | | | |

Supplementary Table 6. Relation of number of ethical statements with study characteristics (*n*=474*)

| **Characteristics** | **Parameter estimates** | **Std.Error** | **Wald** | **Sig** | **95% CI** | |
| --- | --- | --- | --- | --- | --- | --- |
|  |  |  |  |  | **Lower Bound** | **Upper Bound** |
| **Study design** |  |  |  |  |  |  |
| Case report | reference | — | — | — | — | — |
| Case series | 0.929 | 0.199 | 21.876 | **<0.001** | 0.540 | 1.319 |
| **Year** | 0.082 | 0.026 | 9.560 | **0.002** | 0.030 | 0.134 |
| **Sample population** |  |  |  |  |  |  |
| Adults | reference | — | — | — | — | — |
| Children/Infants | 0.265 | 0.265 | 0.995 | 0.319 | -0.255 | 0.784 |
| Both | 0.420 | 0.292 | 2.075 | 0.150 | -0.151 | 0.991 |
| Multinational Research |  |  |  |  |  |  |
| Yes | 0.444 | 0.264 | 2.837 | 0.092 | -0.073 | 0.961 |
| No | reference | — | — | — | — | — |
| **Country continent of first authors** |  |  |  |  |  |  |
| Europe | 0.743 | 0.372 | 3.995 | **0.046** | 0.014 | 1.472 |
| Asia | 0.728 | 0.383 | 3.612 | 0.057 | -0.023 | 1.478 |
| North America | 0.696 | 0.375 | 3.451 | 0.063 | -0.038 | 1.430 |
| Others | reference | — | — | — | — | — |
| **Type of paper** |  |  |  |  |  |  |
| Original | 0.897 | 0.456 | 3.872 | **0.049** | 0.004 | 1.790 |
| Non-original | reference | — | — | — | — | — |
| **Type of study** |  |  |  |  |  |  |
| Clinical | 0.177 | 0.363 | 0.237 | 0.627 | -0.535 | 0.888 |
| Non-clinical | reference | — | — | — | — | — |
| *Link function: Logit.*  *(*) Studies which can not determine sample population or continent of authors, and studies which authors came from mutiple country continents were removed before performing analysis.* | | | | | | |
